# The D‐amino acid transport by the invertebrate SLC6 transporters KAAT1 and CAATCH1 from *Manduca sexta*

**DOI:** 10.14814/phy2.12691

**Published:** 2016-02-16

**Authors:** Alessandra Vollero, Francesca G. Imperiali, Raffaella Cinquetti, Eleonora Margheritis, Antonio Peres, Elena Bossi

**Affiliations:** ^1^ Department of Biotechnology and Life SciencesUniversity of InsubriaVareseItaly; ^2^ Interuniversity Center “The ProteinFactory”Politecnico di MilanoICRM‐CNR Milano and Università dell'InsubriaMilanItaly

**Keywords:** D‐amino acids, SLC6, voltage clamp, *Xenopus* oocytes

## Abstract

The ability of the SLC6 family members, the insect neutral amino acid cotransporter KAAT1(K^+^‐coupled amino acid transporter 1) and its homologous CAATCH1(cation anion activated amino acid transporter/channel), to transport D‐amino acids has been investigated through heterologous expression in *Xenopus laevis* oocytes and electrophysiological techniques. In the presence of D‐isomers of leucine, serine, and proline, the *ms* KAAT1 generates inward, transport‐associated, currents with variable relative potencies, depending on the driving ion Na^+^ or K^+^. Higher concentrations of D‐leucine (≥1 mmol/L) give rise to an anomalous response that suggests the existence of a second binding site with inhibitory action on the transport process. *ms* CAATCH1 is also able to transport the D‐amino acids tested, including D‐leucine, whereas L‐leucine acts as a blocker. A similar behavior is exhibited by the KAAT1 mutant S308T, confirming the relevance of the residue in this position in L‐leucine binding and the different interaction of D‐leucine with residues involved in transport mechanism. D‐leucine and D‐serine on various vertebrate orthologs B^0^ AT1 (SLC6A19) elicited only a very small current and singular behavior was not observed, indicating that it is specific of the insect neutral amino acid transporters. These findings highlight the relevance of D‐amino acid absorption in the insect nutrition and metabolism and may provide new evidences in the molecular transport mechanism of SLC6 family.

## Introduction

KAAT1 (K^+^‐coupled amino acid transporter 1) (Castagna et al. 1998) and CAATCH1 (cation anion activated amino acid transporter/channel) (Feldman et al. 2000), expressed in the midgut and in salivary glands of *Manduca sexta* larvae, belong to the NSS (neurotransmitter sodium symporter) or SLC6 (SoLute Carrier 6) family. These transporters are NATs, nutrient amino acid transporter, classified as B^0^ (the Broad neutral amino acid spectrum system‐(Stevens et al. 1982) for their substrate spectrum). They are able to utilize the Na^+^ and/or K^+^ electrochemical gradients to transport amino acids (Boudko et al. 2005; Boudko 2012). The crystallization of the bacterial homolog LeuT*Aa* represented a keystone to understand the molecular physiology and the structure of the SLC6 family (Yamashita et al. 2005). However, the molecular determinants of the coupling mechanism have not been completely understood, and the differences in functional characteristics among the SLC6 members may be exploited to investigate this aspect. In particular, KAAT1 and CAATCH1 represent an interesting tool, because their transport currents show peculiar properties, such as driving ion dependence, selectivity among transported amino acids, pH, chloride, and membrane voltage dependence (Bossi et al. 1999a, 2007b; Soragna et al. 2004; Castagna et al. 2009).

In this experimental work, we studied the ability of *ms*KAAT1 to transport D‐isomers of amino acids. Our research was suggested by the work of Boudko and coworkers (Miller et al. 2008), who identified the first *Drosophila melanogaster* functionally expressed NAT‐SLC6 member. This transporter showed an unusual broad spectrum of substrates and an extraordinary ability to absorb the D‐isomers of essential amino acids. *Dm*NAT1 is highly expressed in the brain and in the absorptive and secretory regions of the larval alimentary canal, confirming its roles in the absorption and redistribution of neutral amino acids. Internalizing D‐amino acids during the larval stage, *Drosophila melanogaster* can use them in beneficial way in nutrition. This discovery extends the physiological significance of the SLC6 family members.

Because almost all mammalian transporters are selective for L‐amino acids, the D‐amino acids are generally considered unrelated to metabolic pathways. Nevertheless, D‐amino acids were found in many organisms, for example, in the peptidoglycans of bacterial cell wall (Hatanaka et al. 2002), as major components of cellular fluids of some insects and marine invertebrates, in higher plants, in the white matter of human brain (Bauer et al. 2005), in a variety of peptides synthesized by animal cells (include opiate and antimicrobial peptides from amphibian skin, neuropeptides from snail ganglia, a hormone from crustaceans, and a constituent of a spider venom) (Corrigan 1969; Kreil 1997). Furthermore, there are evidences of physiological presence of D‐amino acids in mammalian systems (Man and Bada 1987; Friedman 1999, 2010) and the essential role of D‐serine in central nervous system is well known. This amino acid is produced endogenously in the brain by racemization of L‐serine mediated by serine racemase (Wolosker et al. 1999a,b, 2008; Sacchi 2013), and it is involved in the modulation of glutamatergic neurotransmission by activation of glutamate signaling via the N‐methyl‐D‐aspartate (NMDA) receptor (Mothet et al. 2000).

Different authors reported that insects accumulate significant levels of D‐amino acids from symbiotic sources. In particular, they have the ability to use several D‐forms as essential amino acids during the growth phase, in place of their L‐isomers. Moreover, D‐amino acids play a significant role in invertebrate metabolism and neurotransmission (Geer 1966; Miller et al. 2008; Limmer et al. 2014).

Following these considerations, the presence of D‐amino acid transport currents was investigated in *Xenopus laevis* oocytes expressing *ms*KAAT1 or *ms*CAATCH1 in order to verify their ability to transport D‐isomers, in the presence of NaCl 98 mmol/L or KCl 98 mmol/L. D‐ and L‐isomers of proline, serine (3 mmol/L), and leucine (1 mmol/L) were considered. Leucine was tested at lower concentration to avoid endogenous inward currents sometimes observed with 3 mmol/L in some batches of noninjected (NI) oocytes. The spectrum of substrates was selected according to previous data (Margheritis et al. 2013).

## Methods

Ethical approval: All applicable international, national, and institutional guidelines for the care and use of animals were followed and all procedures performed in studies involving animals were in accordance with the ethical standards of the institution at which the studies were conducted (permit no. 12/09).

### Oocyte preparation and mRNA injection

*Xenopus laevis* oocytes and RNAs were prepared as previously described in detail (Bossi et al. 2007a). Oocytes were obtained from mature female of *X. laevis*; the frogs were anesthetized in MS222 (tricaine methanesulfonate salt; Sigma, Milan, Italy, www.sigmaaldrich.com) 0.10% w/v solution in tap water and portions of the ovary were removed through an incision on the abdomen. The oocytes were treated with collagenase Type A1 (1 mg/mL; Sigma) in calcium‐free solution ND96 for at least 1 h at 16°C. Healthy oocytes were selected and maintained at 16°C in MBS medium (modified Barth's saline solution). After 24 h, oocytes were injected with 50 nL of the in vitro synthesized cRNA (12.5 ng/oocyte) using manual Drummond injection system (Drummond Scientific Company, Broomall, PA, www.drummondsci.com). The oocytes were incubated at 16°C for 4–6 days in MBS before electrophysiological studies.

### Solutions

The oocyte culture and washing solutions had the following composition: calcium‐free ND96: NaCl 96 mmol/L, KCl 2 mmol/L, MgCl_2_ 1 mmol/L, HEPES 5 mmol/L; ND96: calcium‐free ND96 plus CaCl_2_ 1.8 mmol/L; MBS: NaCl 88 mmol/L, KCl 1 mmol/L, NaHCO_3_ 2.4 mmol/L, HEPES 15 mmol/L, Ca(NO_3_) 0.30 mmol/L, CaCl_2_ 0.41 mmol/L, MgSO_4_ 0.82 mmol/L, sodium penicillin 10 *μ*g/mL, streptomycin sulfate 10 *μ*g/mL, gentamicin sulfate 100 *μ*g/mL.

The external control solution used for electrophysiological recording had the following composition: NaCl 98 mmol/L: MgCl_2_ 1 mmol/L, CaCl_2_ 1.8 mmol/L, HEPES 5 mmol/L, pH 7.6. In some conditions, the NaCl was totally substituted by KCl. The final pH of the solutions was adjusted to 7.6 with NaOH and KOH, respectively. The substrates, dissolved in water, were added at the indicated concentrations. Experiments were conducted at room temperature (20–25°C).

### Electrophysiology and data analysis

A two‐microelectrode voltage‐clamp was used to perform electrophysiological experiments (Geneclamp 500B, Axon Instruments, Union City, CA). Intracellular glass microelectrodes, filled with KCl 3 mol/L and with tip resistance between 0.5 and 4 MΩ, were used. Agar bridges (3% agar in 3 mol/L KCl) connected the bath electrodes to the experimental chamber.

The oocytes were clamped at a fixed holding potential of −60 mV (*V*_h_) or a protocol consisting of 1 sec long voltage ramps spanning the range from −140 mV to +40 mV was applied. During the recording, the cells were superfused with external solutions of NaCl 98 mmol/L or KCl 98 mmol/L at pH 7.6 in the presence or in the absence of substrates: L‐ or D‐leucine, L‐ or D‐proline, and L‐ or D‐serine at the concentration indicated in figures ranging from 0.01 mmol/L to 3 mmol/L).Transport currents, reported in the histograms, were determined subtracting the current before the application of the indicated substrate from the current in its presence (mean ± SEM).When ramp protocol was applied transport currents were obtained by subtracting the current level in the absence of substrate (Fig. 5A point *a*) from that recorded in the indicated conditions (point *b, c, d*). Data acquisition and analysis were done using the pClamp 10.2 software (Molecular Devices, Sunnyvale, CA, www.moleculardevices.com), whereas statistics and figures were performed with Origin 8.0 (OriginLab Corp., Northampton, MA, www.originlab.com).

### D‐serine uptake

Control oocytes and those injected with the transporter, clamped at −60 mV, were perfused with D‐serine 300 μmol/L in control solution for 5 min and then washed until the current returned to resting condition. Oocytes were homogenized 1:10 in 0.2 mol/L TCA and centrifuged at 13,000 × *g* for 10 min. Clear supernatants were collected and analyzed with slight modification accordingly to (Topo et al. 2010); supernatants were neutralized with NaOH 0.2 mol/L and subjected to precolumn derivatization with O‐phthaldialdehyde/N‐acetyl‐L‐cysteine (OPA/NAC) in 50% methanol. Diastereoisomers derivatives were then resolved as reported in (Sacchi et al. 2008) on a Simmetry C8 5 *μ*m reversed‐phase column (Waters, 4.6 × 250 mm), in isocratic conditions (0.1 mol/L sodium acetate buffer, pH 6.2, 1% tetrahydrofuran, 1 mL/min flow rate). A washing step in 0.1 mol/L sodium acetate buffer, 3% tetrahydrofuran, and 47% acetonitrile was performed after every single run. Identification and quantification of D‐ and L‐serine was based on retention times (22 ± 0.1 min and 24 ± 0.1 min for D‐ and L‐Ser, respectively) and peaks areas, compared with those associated with external standards. The uptake of D‐serine was calculated for single oocyte in 5 min of perfusion. Oocytes not perfused with D‐serine 300 μmol/L were analyzed to quantify the intracellular level of D‐Serine.

## Results

### D‐amino acid transport with Na^+^ as driving ion

**Figure 1:**
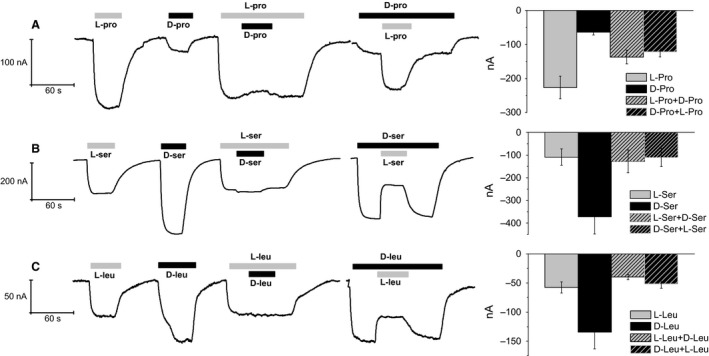
Transport current in the presence of D‐ and L‐ amino acids. Single oocytes (left) and mean values ± SEM for several (*n* = from 5 to 25) oocytes (right). Relative efficiency of L‐ versus D‐ proline 3 mmol/L (A), serine 3 mmol/L (B), and leucine 0.5 mmol/L (C) in eliciting transport‐associated current by KAAT1 when Na^+^ is the driving ion. In the case of proline, the D‐ enantiomer is less potent than the L‐form, whereas the opposite occurs for leucine and serine. In all cases, the L‐form is dominant over the D‐form used at the same concentration: the current amplitude upon simultaneous perfusion of both enantiomers is always close to the level generated by the L‐form alone. Currents were recorded as described in Material and Methods.

Three pairs of L‐ and D‐amino acids, leucine and proline that are classical substrates tested on this transporter and serine that was selected for its role in central nervous system, elicited transport currents in oocytes expressing the wild‐type form of KAAT1 and clamped at *V*_h_ = −60 mV (Fig. 1). In these experiments, the saturating concentration of the L‐form was used for both enantiomers (Miszner et al. 2007). In all cases the D‐forms are able to elicit transport currents. However, the currents generated by the three amino acids exhibit different features: D‐proline is less potent than the L‐form at the same concentration, and addition of 3 mmol/L D‐ to 3 mmol/L L‐proline fails to significantly affect the current amplitude. On the contrary, the addition of 3 mmol/L L‐ to 3 mmol/L D‐proline brings the current amplitude close to the level induced by the L‐form alone (Fig. 1A). An opposite relative potency is exhibited by serine (Fig. 1B). The D‐form is able to generate a much larger current than the L‐form; however, the addition of 3 mmol/L D‐ to 3 mmol/L L‐serine causes only a very weak increase in the current generated by L‐serine, whereas the addition of 3 mmol/L L to 3 mmol/L D‐serine reduces the current level to that of L‐serine alone. The currents generated by the two leucine enantiomers at 0.5 mmol/L are qualitatively similar to those produced by serine, as illustrated in Figure 1C. In all cases, therefore, it appears that the L‐form is dominant over the D‐form when applied at the same concentration, in spite of the different relative potency of the two forms on the size of the current.

### D‐serine uptake

To verify the effective uptake of D‐amino acid by KAAT1 transporter a novel approach was used. Briefly, control oocytes and those injected with the transporter were perfused with D‐serine 300 μmol/L for 5 min at holding potential of −60 mV and then washed until the current returned to resting condition; oocytes were homogenized and the intracellular D‐serine quantified by HPLC. The basal amount of D‐serine in water injected oocytes was 49.72 (±4.41) pmoles/oocyte; when clamped at −60 mV and exposed to 300 μmol/L of D‐serine for 5 min it increased at 61.51 (± 6.74), and in oocytes expressing KAAT1 it was significantly increased, 204.55 (±59.59) pmoles/oocyte (data from 10 oocytes of 2 batches, *P* < 0.05).

### L‐ and D‐leucine dose–response analysis

**Figure 2:**
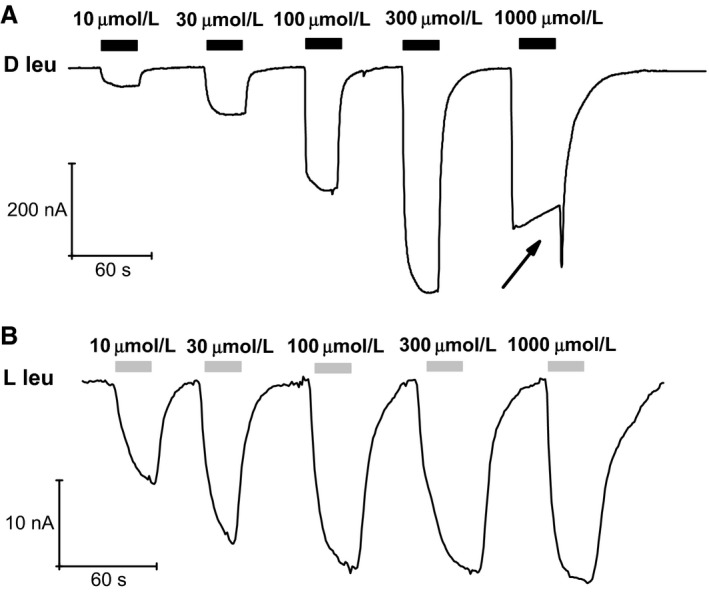
Leucine dose‐response in Na^+^. (A) the transport currents elicited by increasing amounts of D‐leucine show a progressive growth up to 300 μmol/L, but a further increase to 1 mmol/L produces an abrupt change in behavior exhibiting a reduction of the maximum attained value, an inactivation process and a peculiar inward transient surge at washout (arrow). (B) the same amounts of L‐leucine induce progressively larger responses showing saturation above 100 μmol/L without any anomalous behavior.

The dominance behavior described above suggests a lower affinity for the D‐forms compared to the corresponding L‐forms. The apparent affinity of KAAT1 for L‐leucine has been previously determined (Miszner et al. 2007) to be 12 μmol/L at −60 mV. An attempt to determine the affinity of D‐leucine by performing dose–response experiments is shown in Figure 2A. Application of increasing concentrations of D‐leucine to the oocyte caused progressively larger transport currents up to 300 μmol/L. However, when a 1 mmol/L leucine was applied a peculiar behavior was observed: the fast inward initial current soon declined to lower values during substrate exposure, and a marked (and highly reproducible) transient increase in inward current was seen upon substrate washout (arrow).

For comparison, the currents elicited by the same concentrations of L‐leucine are shown in Figure 2B. In this case, no strange behaviors are observed, the current amplitude level was similar for concentrations larger than 100 μmol/L, and the current generated by 10 μmol/L is about half the maximal value, consistent with the previously reported values (Miszner et al. 2007).

Clearly, the peculiar behavior of the current produced by D‐leucine at higher concentrations prevents the determination of the affinity for this enantiomer. However, the large increase in the current elicited by 300 μmol/L compared to 100 μmol/L D‐leucine in the trace of Figure 2A indicates that the apparent affinity of the transporter for the D‐form should be lower than that for the L‐form. Furthermore, the sudden change in the shape of the response between 0.3 and 1 mmol/L D‐leucine may suggest the presence of two different binding sites with different affinities and roles.

To confirm this hypothesis, we exposed KAAT1‐expressing oocytes to mixtures of L‐ and D‐leucine in which the proportion of the two enantiomers varied while keeping their sum constant at a value (500 μmol/L), below the critical level for the appearance of the D‐leucine peculiar behavior.

**Figure 3:**
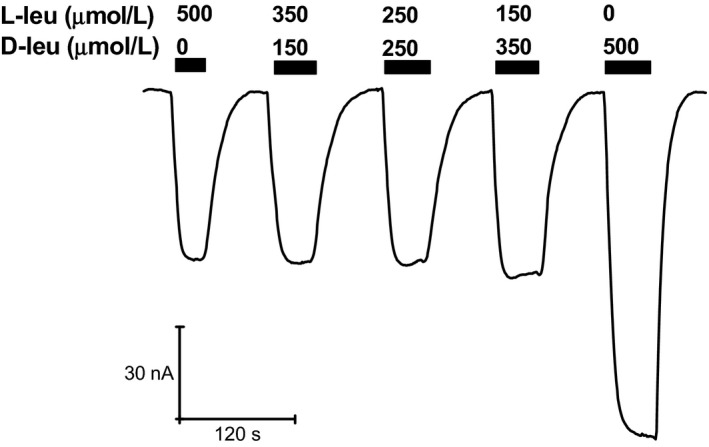
Mole fraction behavior of L‐leu and D‐leu. Mixtures containing variable proportions of L‐ and D‐leucine were applied while keeping constant the total concentration. The substantial constancy of the responses to mixtures containing L‐leucine indicates that this amino acid dominates the transport process by virtue of a higher apparent affinity. The larger current induced by D‐leucine alone is consistent with a higher turnover rate related to a lower affinity. Normalizing the transport current values to that recorded in 500 μmol/L of L‐leucine alone, the current in the presence of 350 μmol/L L‐Leu+150 μmol/L D‐Leu increases to 1.0769 ± 0.01566, when 250 μmol/L of both were perfused becomes 1.13986 ± 0.05827 and when 150 μmol/L L‐Leu and 350 μmol/L D‐Leu was tested the current is 1.20799 ± 0.03927.When 500 μmol/L D‐leucine is applied alone, it becomes from 2 to 4 times larger (mean 2.54265 ± 0.52057) data were collected from 10 oocytes of three different batches.

This kind of experiments is shown in Figure 3 where it is evident that when L‐leucine is present (at ≥150 μmol/L) the transport current remains substantially constant in spite of the presence of complementary amounts of D‐leucine. As already seen in Figure 1, when 500 μmol/L D‐leucine is applied alone, a much larger current is observed. This suggests that up to 500 μmol/L the two forms compete for the same binding site, and that L‐leucine dominates this competition because of its higher affinity for the transporter.

### Anomalous effects of D‐leucine at higher concentrations

**Figure 4:**
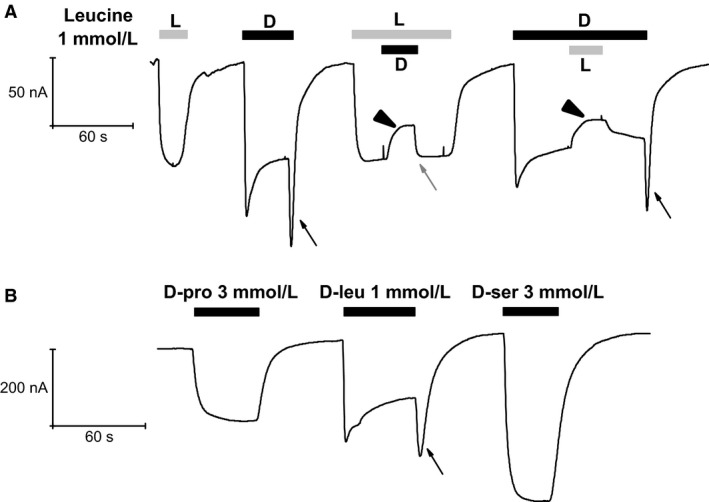
Effects of D‐leucine at 1 mmol/L. (A) when used at 1 mmol/L D‐leucine produces currents with peculiar behavior. A marked decline follows the large and fast initial current during exposure to the substrate and, quite notably, an inward surge of current develops when the substrate is removed (arrows). Note that the surge is not present when the removal of D‐leucine occurs in the presence of L‐leucine (gray arrow). Furthermore, the simultaneous presence of both enantiomers causes a reduction of the current generated by either of the two when applied alone (black arrowheads). (B) subsequent application of the D‐forms of proline, leucine, and serine on the same oocyte shows the specificity of the D‐leucine response.

To study the anomalous response to D‐leucine at higher concentrations, we repeated the competition experiments of Figure 1C, using a 1 mmol/L for both enantiomers. The results are shown in Figure 4A: when the two forms are present together, a reduction in the current elicited by a single form is observed, irrespectively of the application order. Furthermore, the fast inward current surge upon D‐leucine removal is not seen if the wash out occurs in the presence of the L‐enantiomer. The reduction in the transport current seen in Figure 4A upon addition of 1 mmol/L L‐leucine to the already present D‐leucine is analogous to the result shown in Figure 1C where the two substrates were applied at a lower concentration, and can be similarly explained by the higher L‐leucine affinity. Complementarily, no effect should be expected when 1 mmol/L D‐leucine is added to the already present L‐leucine. However, the experiment of Figure 4A shows that a reduction in transport current occurs in this case as well (black arrowheads), also when L‐threonine 1 mmol/L is used instead of L‐leucine (data not shown). This is a further anomaly in the effects of D‐leucine at higher concentration and, together with the inactivating behavior of the transport current and the “off” transient response shown in Figures 2 and 4, requires that a more complex kind of interaction of D‐leucine with the transporter should be envisaged.

The recording of Figure 4B strengthens the peculiar behavior of D‐leucine by comparing the currents elicited by the other two D‐amino acids in the same oocyte.

Overall, the results illustrated in the first four figures indicate that D‐leucine behaves as a normal transported substrate for amounts below about 1 mmol/L, and that at higher quantity it has different kind of actions. The appearance of the new anomalous effects at D‐leucine around and above 1 mmol/L suggests the existence, for this amino acid, of a second binding site, distinct from the main site involved in transport, and with lower affinity and inhibitory action.

**Figure 5:**
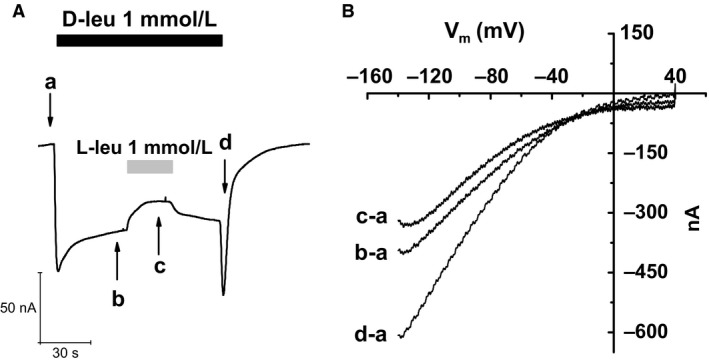
Current–voltage relationship for the different conditions of recording in a representative oocyte. (A) fast voltage ramps (from −140 to +40 mV, duration 1 sec) before the application of substrates (a), in the presence of 1 mmol/L D‐leucine (b), of both D‐ and L‐leucine 1 mmol/L each (c), and upon D‐leucine washout (d). (B) *I*–*V* relationships obtained by subtracting the control current (a) from each of the others.

Regarding the mechanisms through which the anomalous effects of D‐leucine could take place, one possibility is that it might promote the opening of a different conductive pathway in the transporter, in addition to the main transport process. This new current would add up to the transport current, resulting in the peculiar response observed. To test this possibility, we applied fast voltage ramps during different phases of the D‐leu response, including the inward transient at wash out, in order to obtain *I*/*V* relationships in the different conditions. The results, shown in Figure 5, indicate the usual inward‐rectifying shape of the transport currents in all conditions, without any hint for the presence of other current components with different voltage dependence. These data suggest therefore that the current changes seen in all phases of the experiment, including the inward transient upon removal of D‐leucine from the bath, represent real alterations in the activity of the transporter.

### D‐amino acid transport with K^+^ as driving ion

**Figure 6:**
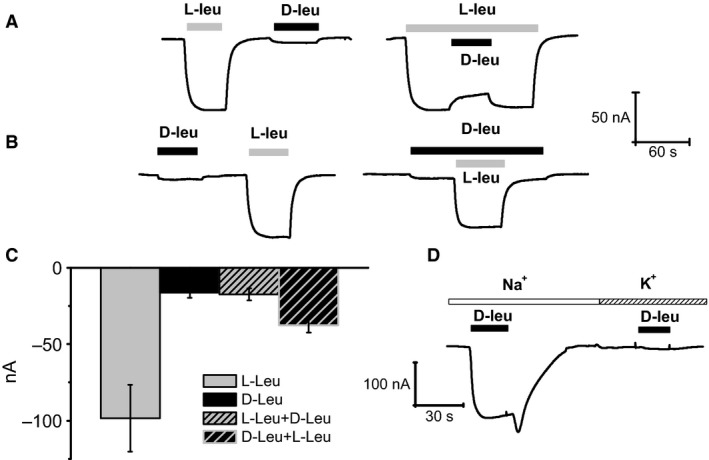
D‐leucine in high K^+^ solution. When applied at 500 μmol/L in a K^+^‐containing external solution, D‐leucine elicits a small current compared to L‐leucine. The addition of D‐ to L‐leucine (both at 500 μmol/L) slightly inhibits the L‐leucine‐induced current (A). The increase in L‐leucine current when applied in addition to D‐leucine (both at 500 μmol/L), does not reach the level elicited by L‐leucine alone (B). The mean values ± SEM for several (*n* = from 6 to 15 from at least 3 different batches) oocytes are reported (C).Comparison of the currents generated by 1 mmol/L D‐leucine in presence of high external Na^+^ or high external K^+^ revealed that, in the latter condition, only a very small transport current is detected (D).

Since KAAT1 is known to be able to use also the K^+^ electrochemical gradient for amino acid transport (Castagna et al. 2009), we measured the transport currents elicited by D‐amino acids in the presence of high external K^+^ concentrations. As shown in Figure 6, 500 μmol/L D‐leucine generates only a small transport current in the presence of high potassium compared to the large response to L‐leucine at the same concentration. When applied together, D‐leucine causes a reduction in the current elicited by the L‐isoform and, conversely, L‐leucine increases the current generated by the D‐isoform, however, to a lesser level than when applied alone. The relative potency of the two stereoisomers is opposite to that observed in Na^+^, and the results of the competition experiments are analogous to those seen in Na^+^ for proline. Increasing the D‐leucine to 1 mmol/L does not cause any anomalous behavior, as seen in Figure 6D, where the currents generated in Na^+^ and in K^+^ are compared.

**Figure 7:**
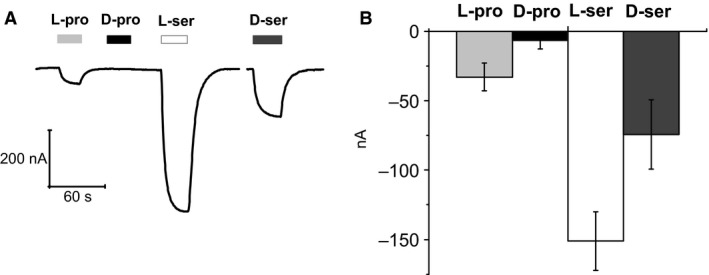
KAAT1, currents elicited by D‐proline and D‐serine in K^+^. L‐proline 3 mmol/L elicits only small transport currents in presence of K^+^, whereas D‐proline at the same concentration appears to be completely ineffective. On the contrary, both L‐ and D‐serine 3 mmol/L generate currents in K^+^, although with opposite relative potency compared to those in Na^+^ (A).The mean values ± SEM for several oocytes (*n* = from 6 to 15 from at least 3 different batches) are reported (B).

We also tested D‐proline and D‐serine in the presence of high external K^+^. The results, shown in Figure 7, indicate that the already small transport current elicited by L‐proline vanishes in the case of the D‐enantiomer. Regarding serine, a strong current is elicited by the L‐form, whereas the D‐form generates a smaller current. Interestingly, this result is the opposite of the observation in Na^+^, where D‐serine gives the larger current.

### D‐amino acid transport in CAATCH1

**Figure 8:**
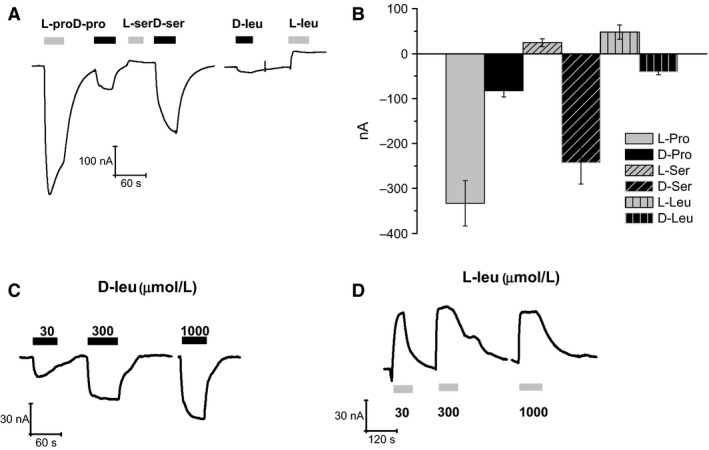
Action of different L‐ and D‐amino acids on the CAATCH1 transport currents in Na^+^. (A and B) L‐proline is more potent than the D‐form in eliciting the transport current; L‐serine and L‐leucine generate an apparent outward current, while their corresponding D‐forms produce an inward current. (A) a representative recording; (B) mean current  ± SEM from 6 to 11 oocytes from two different batches. (C) application of increasing concentrations of D‐leucine produces progressively larger inward currents, whereas the same concentrations of L‐leucine produce an apparent outward current of constant size (D), consistent with a high‐affinity block of the transporter leak current (Miszner et al. 2007).

The *Manduca sexta* intestine contains another transporter, CAATCH1 that is structurally related to KAAT1, but shows dissimilar substrate selectivity. Noticeable is the observation that L‐leucine acts as a blocker rather than a transported substrate (Miszner et al. 2007). Figure 8A and B shows a sequence of current responses to various L‐ and D‐amino acids in a CAATCH1‐expressing oocyte bathed in high Na^+^ solution. As already observed (Soragna et al. 2004), L‐proline elicits large transport currents, whereas L‐leucine produces an apparent outward current, actually representing a block of the leak current through the transporter. The corresponding D‐enantiomers produce a reduced (D‐proline) or a small inward current (D‐leucine). L‐serine, which was not previously tested in this transporter, has a blocking effect similar to that of L‐leucine, whereas the D‐enantiomer gives rise to a large current.

The results of Figure 8A indicate that in CAATCH1 L‐ and D‐proline behave qualitatively in the same way as in KAAT1, and also that the serine and leucine enantiomers behave similar to each other, both the L‐forms producing apparent outward currents. Application of D‐ and L‐leucine at different concentrations (range between 30 and 1000 μmol/L, Fig. 8C and D) demonstrates the different nature of the current changes generated by the two substrates. The amplitude of the response to D‐leucine increases with the substrate concentration, indicative of an existent transport current, whereas the identical level reached by the responses to L‐leucine is consistent with a high‐affinity block of the transporter leak current, as previously suggested (Miszner et al. 2007).

Furthermore, no anomalous behavior is detected in Figure 8C upon application of 1 mmol/L D‐leucine on CAATCH1.

### D‐amino acid transport in the KAAT1 mutant S308T

**Figure 9:**
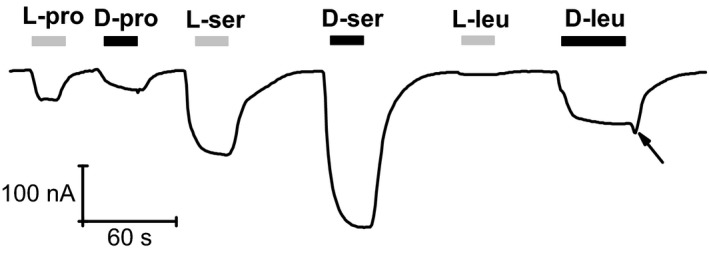
Currents elicited by L‐ and D‐amino acid in KAAT1 S308T. While the currents generated by serine 3 mmol/L, and their relative potency, are not affected by the mutation, a selective reduction of the current of the L‐form is seen for proline 3 mmol/L. As expected, no current is generated by L‐leucine 1 mmol/L, whereas the same concentration of D‐leucine is effective in producing a response that includes a weaker but reproducible inward current surge at wash out (arrow).

We have previously shown (Miszner et al. 2007) that a single‐point mutation (S308T) in KAAT1 selectively abolishes L‐leucine transport. We have then checked whether the transport of D‐leucine is also affected in this mutant. Figure 9 shows that this is not the case and, furthermore, that the peculiar behavior of the current elicited by 1 mmol/L D‐leucine remains, although its distinctive features appear somehow reduced (slower initial development of the current, no inactivation, small inward transient at washout). The abolition of the L‐leucine current is confirmed, the relative potency of the serine enantiomers is conserved as well, whereas the current induced by L‐proline is strongly reduced in comparison to the wild type, reaching a level similar to the D‐isoform.

## Discussion

Up to now, all functional investigations on KAAT1 have been focused on the transport of L stereoisomers of neutral amino acids (Castagna et al. 1998; Bossi et al. 1999a,b; Miszner et al. 2007; Giovanola et al. 2012) and in other species as well, only few studies have considered the ability of membrane transporters to absorb the D‐isoforms. About insect transporters Miller (Miller et al. 2008) has shown that *Dm*NAT1, a *D. melanogaster* transporter homologous to KAAT1, is able of transporting L‐ and D‐isomers of several amino acids with similar efficiency. D‐amino acids are common components of the bacterial cell wall; they are produced by the normal metabolism of yeast and bacteria and can be found in extracellular fluids of insects and marine invertebrates. As both *Dm*NAT1 and *ms*KAAT1 are highly expressed in the gut epithelium, it is reasonable to hypothesize that these insects might feed with products derived from bacterial fermentation and symbiosis. Similar investigation on various vertebrate orthologs B^0^AT1 (SLC6A19) showed that D‐leucine and D‐serine elicited only a small current (less than 10% of the current generated by the corresponding L‐forms) and they failed to block the transport current and peculiar behavior was not observed, underlining that observations reported in this study are specific of the insect neutral amino acid transporters.

### Relative potency of L‐ and D‐ isoforms transport in KAAT1

The results of the electrophysiological experiments have shown that *ms*KAAT1 is able to transport D‐amino acids with variable relative potencies with respect to the corresponding L‐isoform, depending on the amino acid and on the driving ion. In fact, as shown in Figure 1, when the driving ion is Na^+^ the amplitude of the transport currents generated by the D‐forms of serine and leucine are much larger than that of the corresponding L‐forms, whereas in the case of proline the opposite is true. The D‐serine detection by HPLC of the uptake of this D‐amino acid under voltage clamp confirmed the actual entrance of D‐serine and the uptake amount is in agreement with previous data recorded for this transporter (Bossi et al. 2000).

When the driving ion is K^+^ the responses to the L‐forms of serine and leucine are definitely more potent than those of the D‐forms and, in the case of proline, the D‐form appears unable of eliciting transport currents (Figs. 6 and 7). This variability is not surprising since ion‐dependent differential potency orders were already observed among various L‐amino acids (Soragna et al. 2004; Miszner et al. 2007), and are very likely due to the different molecular interactions that the various substrates may establish with the transporter binding site in the presence of Na^+^ or K^+^.

Although it is difficult to establish what are the normal physiological concentrations of sodium and potassium in the gut of the larva, the fact that in both ionic conditions some D‐amino acids may be absorbed via the transporter suggests that the insect might indeed obtain nutrients from the products of bacterial fermentation (digestion) in a wide range of ionic conditions.

### Competition experiments

To understand the relations between the two enantiomers of each amino acid and the transporter, we performed competition experiments in which the currents elicited by each enantiomer alone were compared to the current generated when both were present together at the same concentration. The results at lower concentrations, shown in Figure 1, indicate that for the three tested amino acids, addition of the D‐form does not significantly alter the current amplitude generated by the already present L‐form. While, when the application order is reversed, the addition of the L‐form increases (for proline), or reduces (for serine and leucine) the current level generated by the D‐form alone, bringing the amplitude to the level corresponding to the L‐form alone. The conclusion from these experiments is that in all the three cases the L‐forms are dominant over the D‐forms, very likely because their affinity for the transporter is higher.

A dominance effect of this kind, namely of L‐leucine with respect to L‐proline and L‐threonine was already observed in KAAT1 and ascribed to the much higher affinity of the transporter for leucine (Miszner et al. 2007).

Indeed, although the determination of the apparent affinity for D‐leucine was not possible because of the anomalous response arising at higher substrate concentrations, the data of Figure 2 clearly indicate that the apparent affinity of D‐leucine for the transport process is lower than that of L‐leucine. A lower affinity for the transport process is consistent with a higher turnover rate, and consequently with a larger transport current, as already observed when comparing different L‐amino acid transported by KAAT1 (Miszner et al. 2007), and as seen in the present work for L‐ and D‐leucine in the presence of Na^+^.

### Anomalous effects at high D‐leucine concentrations

As described in detail in the Results section, an abrupt change in the shape of the response occurs when the D‐leucine concentration is increased from 0.3 to 1 mmol/L: the peak of the inward current is reduced compared to that of the lower concentrations, the currents shows a slow inactivation, and a conspicuous surge of inward current appears upon washout of the amino acid. This behavior is peculiar to D‐leucine, as higher than 0.3 mmol/L of D‐serine or D‐proline fail to elicit such kind of response (Fig. 4B). Another remarkable effect of D‐leucine at 1 mmol/L is apparent when performing competition experiments: as shown in Figure 4A, and in contrast to the result of Figure 1C, the addition of 1 mmol/L D‐leucine significantly decreases the transport current generated by 1 mmol/L L‐leucine alone. Furthermore, it is interesting to note that when D‐leucine is washed out while in the continued presence of L‐leucine, no transient surge of inward current is observed (Fig. 4A gray arrow).

This series of peculiar observations requires envisioning some new kind of interaction between D‐leucine and the transporter. The existence of a second binding site for D‐leucine with an affinity lower than that of the site involved in the transport mechanism is suggested by the fact that all the odd effects arise sharply when the amino acid concentration reaches a value of about 1 mmol/L. As no evidences for the existence of conductive pathways different from the transport process are given by the current–voltage relationships shown in Figure 5, a remaining possibility is that the second binding site should exert an allosteric inhibition on the transport process itself. The typical time course of current elicited by 1 mmol/L D‐leucine might then be explained as follows: due to the inevitable delay of the perfusion system, initially the substrate concentration in proximity of the cell activates the transport process generating the inward current; when the substrate concentration is sufficient to bind to the second – inhibitory – site, the current stops increasing and a slower decline begins; when the application of the substrate is ended, its concentration falls, the inhibitory site is soon voided and a surge of inward current occurs because for a while there is still enough substrate to be transported.

This hypothesis can also explain the apparent anomalies observed in the competition experiment of Figure 4A: the current reduction resulting from the addition of D‐leucine to the already present L‐leucine is caused by the binding to the inhibitory site, whereas the absence of the inward surge of current when D‐leucine is washed in the continuous presence of L‐leucine is due to the lower turnover rate for this latter substrate.

### D‐amino acid transport in CAATCH1 and in the KAAT1 mutant S308T

Previous studies (Soragna et al. 2004; Miszner et al. 2007) have shown that CAATCH1, a KAAT1 homologous also present in the *Manduca sexta* intestine, is not able to transport L‐leucine. The experiments such as that shown in Figure 8 indicate that also CAATCH1 is able to transport D‐amino acids with relative potencies qualitatively analogous to those observed in KAAT1. A notable difference is the lack of transport current upon application of L‐serine in contrast to the large response to the D‐enantiomer. These findings support the possibility that CAATCH1 may represent an additional route for D‐amino acids absorption in this insect species.

The blocking action of L‐leucine on the leak current of CAATCH1 is due to the inability to proceed along the transport cycle after substrate binding. A similar effect is seen in a KAAT1 mutant in which, the serine in position 308, a critical residue for leucine binding, is replaced by the threonine present in the same position in CAATCH1 (Miszner et al. 2007). Our experiments with this mutant (Fig. 9) show that the ability to transport L‐ and D‐enantiomers of serine and proline is qualitatively unchanged compared to the wild type. However, while the inability to transport L‐leucine is confirmed, S308T is still able to transport D‐leucine. The response to D‐leucine 1 mmol/L does not show the strikingly odd features described in the wild type, although a small inward surge of current is still visible at substrate washout. These results suggest that the affinity of the primary binding site for both L‐ and D‐leucine is significantly increased in the mutant, abolishing the L‐leucine response, and blunting the response to D‐leucine. The presence of the inward surge at washout indicates that the second, inhibitory, site is still operating.

### Nutritional and functional relevance of D‐amino acid absorption

The membrane transporters belonging to the NAT family are mainly located in the intestinal epithelium and in neurons and glial cells (Boudko et al. 2005; Broer et al. 2005; Takanaga et al. 2005; Meleshkevitch et al. 2006). Their capacity to absorb D‐amino acid is therefore relevant not only for the nutritional aspect in general, but also for the needs of amino acids in the central nervous system. This suggests a possible role of these transporters in delivering substrates for the synthesis of neurotransmitters, or to provide the brain with D‐serine, an important modulator of the glutamatergic neurotransmission.

## Conflict of Interest

None declared.
